# Child body mass index in four cities of East China compared to Western references

**DOI:** 10.1080/03014460802575641

**Published:** 2008-12-10

**Authors:** Huiqi Pan, Yifang Jiang, Xinming Jing, Sulin Fu, Yan Jiang, Zhongfang Lin, Zhihua Sheng, Tim J. Cole

**Affiliations:** 1 MRC Centre of Epidemiology for Child Health, UCL Institute of Child Health, UK; 2 Shanghai Children's Hospital, Jiao Tong University, Shanghai, China; 3 Shanghai Children's Medical Centre, Jiao Tong University, Shanghai, China; 4 Hefei Institute of Maternal and Child Health, Hefei, China; 5 Central Hospital of Jinan, Jinan, China; 6 Xuzhou Maternal and Child Health Care Centre, Xuzhou, China

**Keywords:** Child, overweight, obesity, body mass index, China

## Abstract

**Background:**

The rising trends in child obesity worldwide are poorly documented in China.

**Aim:**

The present study compared the distribution of body mass index (BMI) by age in children from four cities in East China with Western references.

**Subjects and methods:**

94 370 boys and 90 048 girls aged 0–19 years from Shanghai, Jinan, Xuzhou and Hefei were measured in 1999–2004 for length/height and weight. The LMS method was used to construct BMI centiles for each city. Shanghai children aged 0–6 years in 1986 and US and UK BMI references were used for comparison.

**Results:**

The median BMI curves for the four cities differed in shape from those for the USA and UK. Chinese boys were fatter than US boys in early to mid-childhood but less so in adolescence, and US boys were fatter at age 18. Within China the adiposity rebound was earlier in boys than girls. Shanghai children were appreciably fatter in 2000 than in 1986, and boys more so than girls.

**Conclusions:**

The roots of child obesity lie in early life, particularly in boys, and are linked to economic development, which has important implications for both the aetiology of child obesity and the health of current and future Chinese children.

## Introduction

In recent decades the prevalence of child obesity has risen steeply in most parts of the world (Wang and Lobstein 2006; Lobstein and Jackson-Leach 2007). China is a developing country whose economy has been expanding rapidly over the past two decades, and the rates of child obesity in China are less well documented than elsewhere. Luo and Hu (2000) described time trends of obesity based on body mass index (BMI) in Chinese pre-school children from 1989 to 1997; Wang et al. (2002) documented trends of overweight in school children aged 6–18 in 1991 and 1997, and Li et al. (2008) have monitored trends from 1982 to 2002 in children aged 7–17. In addition several articles have given rates of overweight and obesity in Chinese children of different ages based on the International Obesity TaskForce (IOTF) cut-offs of Cole et al. (2000) (Hui and Bell 2003; Liu et al. 2007; Li et al. 2008). The findings have generally been consistent in that overweight and obesity rates have been rising steeply over time, but with the rises restricted to urban areas with no or little increase in rural areas, and boys are consistently fatter than girls. China's economic growth has obviously had an impact on child obesity, but how and to what extent is still unclear. This study aimed to investigate the whole distribution of BMI by age, rather than relying on summary prevalence rates of overweight and obesity, in urban children aged 0–18 years from four cities in Eastern China. A secondary aim was to compare the results for the four cities with each other and with those for Western BMI references.

## Subjects and methods

Shanghai, Jinan, Xuzhou and Hefei are four cities from the Middle East of China, which differ in terms of geographical location and economic growth. Between 1999 and 2004 weight and length/height for children aged 0–19 years were collected from the four cities, using a cluster design, with sample sizes of 96 104, 27 244, 30 007 and 31 063, respectively. The sex ratio boys:girls was 1.048. Between 3% and 5% of the target populations in the four cities were included. The measurements were abstracted from the records of the routine health examinations that take place at 1, 2, 4, 6, 9, 12, 18 and 24 months of age for infants, and twice a year for nursery and school children. Supine length was measured in those under 3 years using a length board, and height over 3 years using a stadiometer. Weight was measured with a lever-arm scale. All instruments were of Chinese origin, and were calibrated regularly. Observers were trained and supervised by the Centres for Disease Control and Prevention (CDC) in the cities.

In China ethical permission is not formally required for routine height and weight measurement. However the programme was supported financially by the Shanghai Municipal Health Bureau and the Shanghai Public Health Bureau, which implies ethical permission. Similarly, permission was obtained from the relevant institutes and hospitals in the other three cities.

Body mass index (BMI = weight (kg)/height (m)^2^) was derived, and growth references for BMI by sex were constructed using the data for the four cities separately. The final BMI references were truncated at 18 years to avoid edge effects. Measurements were converted to *z*-scores (see below), and those with *z*-scores exceeding 5 in absolute value were excluded as outliers from the final dataset – 0.13% of the data were excluded for this reason.

Data for 1112 boys and 1069 girls aged 0–6 years from five districts of urban Shanghai collected in 1986 (Shanghai 1986) were used to produce centiles separately for the comparison (WHO Collaborating Centre for Physical Growth and Psychosocial Development of Children in China 1991). Table I shows the numbers of subjects by age in each city sample.

**Table I:** Sample size of BMI by age.

Age group	Jinan	Xuzhou	Hefei	Shanghai 2000	Shanghai 1986
0–	3941	4025	4216	13 454	861
1–	474	1353	2433	4051	454
2–	372	1451	1593	4063	224
3–	949	1467	1673	3776	215
4–	1081	1217	1416	5371	215
5–	1223	877	1375	5224	212
6–	2669	1250	1165	3970	
7–	1036	1409	992	3733	
8–	1025	1020	1036	4390	
9–	2263	1592	1098	5927	
10–	1232	1311	1084	5962	
11–	710	835	1218	6006	
12–	871	1349	1635	3986	
13–	1176	2337	1718	3952	
14–	1204	2593	1629	4245	
15–	2267	1650	2281	4666	
16–	1430	1153	2130	4227	
17–	1640	1614	1437	3634	
18–19	1681	1404	934	5467	
Total	27 244	30 007	31 063	96 104	2181

The data were analysed by the LMS method, for each city separately by sex (Cole and Green 1992). A Box–Cox power transformation was used to normalize the data at each age. Natural cubic splines with knots at each distinct age *t* were fitted by maximum penalized likelihood to create three smooth curves: *L*(*t*), the Box–Cox power; *M*(*t*), the median; and *S*(*t*), the coefficient of variation.

Centile curves at age *t* were then obtained as

(1) $$C_{100\alpha }(t)=M(t){\left[1+L(t)S(t)Z_{\alpha }\right]}^{1/L(t)}$$

where *Z*_α_ is the normal equivalent deviate for tail area α, and *C*_100α_(*t*) is the centile corresponding to *Z*α. Equivalent degrees of freedom (edf) for *L*(*t*), *M*(*t*) and *S*(*t*) measure the complexity of each fitted curve. *Q* tests (Royston and Wright 2000; Pan and Cole 2004) were used to check the goodness of fit. Inverting equation (1) expresses a child's BMI as a *z*-score:

$$z=\frac{{\left(BMI/M\right)}^{L}-1}{L\times S}$$

where the values for *L*, *M* and *S* are for the child's age and sex.

During the fitting process the age scale was transformed to age^0.2^ for the four cities data, and to age^0.3^ for the Shanghai 1986 data, to improve the fit at young ages. The chosen edf for the *L*, *M* and *S* curves were 6, 12, 6 for Shanghai 2000, and 3, 6 and 3 for others. Table II gives the proportions of data in the channels round the seven fitted centiles of 3rd, 10th 25th, 50th, 75th, 90th and 97th for the data, confirming a good fit.

**Table II:** Distribution of z-scores of BMI for the cities compared to expectation assuming normality – area between adjacent centiles (%).

	Expected	Jinan		Xuzhou		Hefei		Shanghai 2000		Shanghai 1986	
						
Centile	(%)	Boys	Girls	Boys	Girls	Boys	Girls	Boys	Girls	Boys	Girls
3	3	2.6	2.8	2.7	3.1	2.8	3.1	2.6	2.8	2.7	2.8
5	2	1.8	2.0	1.6	1.7	1.7	1.8	1.8	1.8	0.5	1.7
10	5	4.6	4.4	4.9	4.7	4.7	4.7	5.0	4.7	5.1	3.4
25	15	16.5	14.8	15.9	14.8	15.8	14.6	16.1	15.4	15.5	15.7
50	25	26.0	27.2	26.0	26.3	25.8	25.6	25.7	25.8	27.1	26.8
75	25	23.1	24.4	23.6	25.1	24.4	25.8	23.5	24.8	25.9	26.5
90	15	14.0	13.9	14.3	14.2	14.1	14.8	14.7	14.6	15.0	13.3
95	5	5.7	4.9	5.3	5.0	5.4	4.3	5.5	4.8	3.8	5.2
97	2	2.6	2.3	2.6	1.7	2.1	1.9	2.3	2.0	1.2	1.9
100	3	3.1	3.5	3.1	3.4	3.2	3.3	3.0	3.3	3.2	2.8

The national BMI references US CDC 2000 (US) (Kuczmarski et al. 2002) and British 1990 (UK) (Cole et al. 1990) were used for comparison.

Here the BMI distributions by age in the different regions are compared by focusing on centile curves for thin, median and fat children, using the 2nd and 91th centiles as the extremes (corresponding to *z*-scores −2, 0 and +1.33). We justify this asymmetric pattern as follows: The international cut-offs for child overweight (Cole et al. 2000) and thinness (Cole et al. 2007) are commonly used for international comparisons of fatness and thinness, and they correspond broadly to the 91st and 2nd centiles of the British 1990 reference (Cole et al. 1990; Cole 1994). Thus this choice of centiles summarizes a range of adiposity similar to that represented by the international cut-offs.

The international cut-offs (Cole et al. 2000) were also used to compare the prevalence of overweight (excluding obesity) and obesity in the Chinese cities with Health Survey for England data in 2001–2002 (Sproston and Primatesta 2003), representing a more contemporaneous sample than the BMI reference of 1990.

The ‘adiposity rebound’ is the second rise in BMI that occurs between 2 and 7 years (Rolland-Cachera et al. 1984). The summary ages at adiposity rebound for each sample were read off the individual fitted centile curves.

## Results

**Figure 1:**
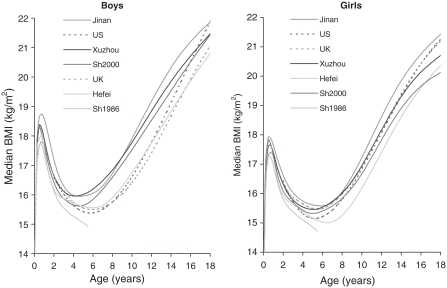
Median BMI in boys and girls by age. Jinan, Xuzhou, Shanghai 2000; Hefei, Shanghai 1986 (solid lines), US CDC 2000 and British 1990 (dashed lines). To help distinguish between the curves, the ordering of the legend matches the ordering of the curves at age 18 (age 6 for Shanghai 1986), so that for example Jinan is both the highest curve at age 18 and the top entry in the legend.

Figure 1 shows median BMI by sex and region (i.e. the four cities and two international references) from birth to 18 years. To help distinguish between the curves, the ordering of the legend matches the ordering of the curves at age 18 (age 6 for Shanghai 1986), so that for example Jinan is both the highest curve at age 18 and the top entry in the legend. The US and UK curves are shown as dotted lines, and it is clear that their relation to the Chinese curves (solid lines) is different for boys and girls. For boys the US and UK curves are appreciably lower after age 5, while for girls the two sets of curves are broadly similar at all ages. Thus the age at adiposity rebound in Chinese boys is about a year earlier than in Western boys or Chinese girls. Yet despite this, BMI at age 18 is similar in boys from China and the West. Also, in both sexes, BMI after 10 years increases with age more slowly in China than the West.

Among the Chinese cities Hefei stands out as having the lowest BMI and the latest adiposity rebound. This applies to both sexes, although by age 18 BMI in Hefei girls is slightly higher than in Shanghai.

Within Shanghai, the 1986 curves (which stop at age 6 in Figure 1) are appreciably lower at all ages and both sexes than in 2000.

**Figure 2:**
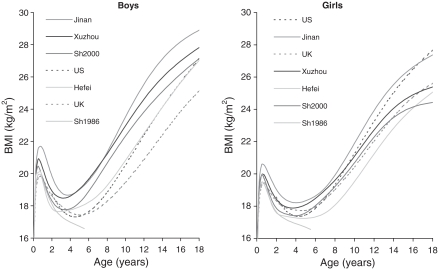
The 91st centiles of BMI in boys and girls by age. Jinan, Xuzhou, Shanghai 2000; Hefei, Shanghai 1986 (solid lines), US CDC 2000 and British 1990 (dashed lines). To help distinguish between the curves, the ordering of the legend matches the ordering of the curves at age 18 (age 6 for Shanghai 1986).

Figure 2 gives the 91st centiles of BMI by age, sex and region. They are further apart than the medians (Figure 1), despite the *y*-axis being stretched, but in terms of age trends and rankings the two Figures are similar. Chinese boys (excepting Shanghai 1986) are still higher than those in the West, and have an earlier adiposity rebound, but the US and UK curves are relatively higher. Chinese girls are broadly similar to the West.

**Figure 3:**
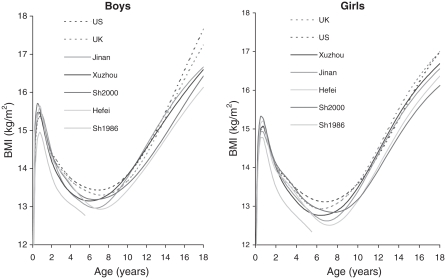
The 2nd centiles of BMI in boys and girls by age. Jinan, Xuzhou, Shanghai 2000; Hefei, Shanghai 1986 (solid lines), US CDC 2000 and British 1990 (dashed lines). To help distinguish between the curves, the ordering of the legend matches the ordering of the curves at age 18 (age 6 for Shanghai 1986).

Figure 3 gives the 2nd centiles of BMI by age, sex and region. Note that the centiles are much closer together, with a *y*-axis range of only 6 BMI units compared to 8 for the medians (Figure 1) and 14 for the 91st centiles (Figure 2). Even taking this into account, the cities are all very similar except for Shanghai 1986, which is thinner. The Western references are higher at all ages in both sexes, although their ages at adiposity rebound are similar.

**Figure 4:**
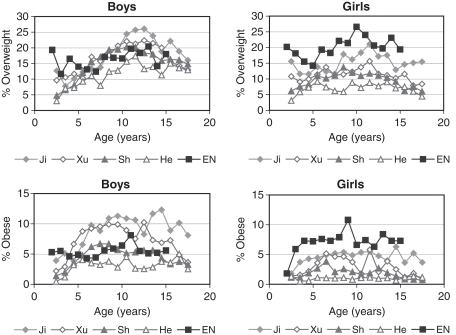
Rates of overweight and obesity in boys and girls by age based on the IOTF cut-offs (Cole et al. 2000) in Jinan, Xuzhou, Shanghai 2000 and Hefei, and the Health Survey for England 2001–2002 (Sproston and Primatesta 2003).

The Chinese data, collected in 1999–2004, are more recent than the US CDC (1963)–1980) or UK 1990 (1978–1993) reference data, so one would expect the Chinese rates to be higher given the upward trends in overweight worldwide. For a more valid comparison Figure 4 compares the prevalence of overweight and obesity in the Chinese data with England in 2001–2002 (Sproston and Primatesta 2003) using the IOTF cut-offs (Cole et al. 2000).

The English rates were relatively constant with age, and slightly higher in girls than boys. The Chinese rates were consistently lower in early life in both sexes, but in boys they rose steeply with age, so that in Jinan and Xuzhou they exceeded those in England for much of childhood, particularly for obesity. Rates declined after adolescence. In girls the rates were consistently lower than in England. In general, overweight and obesity prevalence was higher in boys than girls in China and vice versa in England.

## Discussion

Childhood obesity is a key risk factor for later obesity, type 2 diabetes and hypertension (Alison et al. 2005), and its rising prevalence needs to be documented in China as elsewhere. At the same time we can increase our understanding of the aetiology of child obesity by comparing the different developmental paths of BMI in China and the West.

There are four main findings to emerge from the study: (i) the pattern of age change in BMI through childhood differs between China and the West; (ii) within China this pattern differs between the cities; (iii) Chinese boys are appreciably fatter than Chinese girls, and (iv) in Shanghai, BMI increased steeply between 1986 and 2000.

Chinese boys have a relatively early adiposity rebound, a year earlier in Jinan, Xuzhou and Shanghai than in the US/UK, but BMI subsequently increases more slowly with age. The net effect is a rise then fall in overweight prevalence with age as seen in Figure 4, comparing the Chinese with contemporaneous English data. Conversely Chinese girls have their rebound at much the same age as in the West, but they also slow afterwards and end up thinner.

Chinese children are appreciably thinner in Hefei than the other cities. This may partly be explained by differences in economic growth between the cities. In 2001 the gross domestic products in Hefei, Xuzhou, Jinan and Shanghai were 5.0, 9.6, 14.5 and 54.9 US$ million, respectively, with per capita incomes of 824, 921, 1157 and 1414 US$ (Bean 2003; National Bureau of Statistics of China 2001). Thus Hefei, with the thinnest children, also has the lowest mean family income. Against this Shanghai children, where income is highest, are no fatter than those from Xuzhou or Jinan. This may reflect higher education levels in Shanghai.

Geographical location may also be relevant. Jinan, Xuzhou, Shanghai and Hefei are at latitude 36°50′, 34°16′, 31°52′ and 31°10′ North, respectively, while Shanghai is longitude 121°28′ East as against 117°00′–14′ East for the other cities. Thus Jinan and Xuzhou are 250 and 550 km north of Hefei, while Shanghai is 400 km east of Hefei. People living in the north have longer winters and less opportunity for outdoor activities than those living in the south. This study found that children in the more northern cities are fatter than those in the south, which is consistent with another recent study (Gu et al. 2005).

In China in 2000, boys were appreciably fatter than girls after age 3 and had an earlier adiposity rebound. This may reflect the extra attention that Chinese boys tend to receive in early life compared to girls, where boys are expected to be strong while girls should be slim. The one-child family policy, which started in 1979 (Wang et al. 2002; Potts 2006), may or may not have exacerbated this tendency. However there was no sex difference in BMI in 1986, which suggests that economic growth over the intervening period may also have played a part – see below.

In the West parental obesity is a strong risk factor for child obesity (Reilly et al. 2005; Wardle et al. 2008), and many parents are fat (Flegal et al. 1998). China differs from the West in that adults have traditionally not been fat, although this is rapidly changing (Wu 2006). So fatness in Chinese boys must come from their environment to a greater extent than in the West, and this is a potentially interesting research focus. China can be used as a model to clarify the contributions of on the one hand parental genetics and epigenetics, and on the other hand the child's own early environment, in the development of child obesity.

The fall-off in BMI after puberty in China in 2000 compared to the West may reflect recent trends in Chinese economic development. The oldest children, born in the early 1980s, did not experience rising living standards until later in childhood, whereas those born more recently were exposed at a younger age. This suggests that overweight will increase as these later-born children become adults. It also suggests that early childhood is an important period for later overweight (Baird et al. 2005).

**Figure 5:**
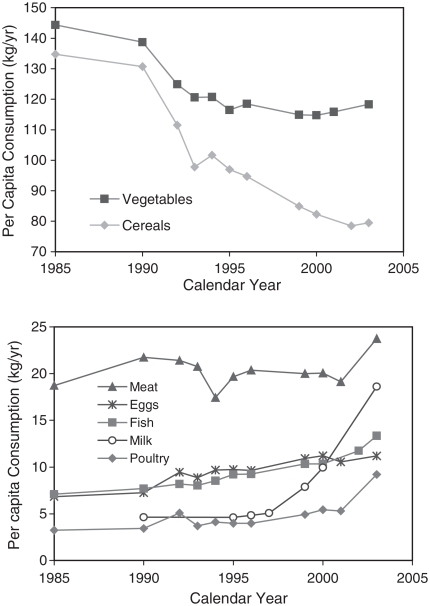
Per capita annual purchases of cereals, vegetables, meat, eggs, fish, milk and poultry of urban households in China by year.

One consequence of China's economic growth has been the change in diet and lifestyle. Children now spend more time watching TV (Wang et al. 2002) or playing computer games than playing outdoors. Many people in cities have moved from central low-rise houses to outlying high-rise flats, and now rely on taxis, buses and trains rather than bicycles to get about, which reduces physical activity. Western-style food has become increasingly available in the past two decades. The Chinese now consume fewer staple foods, and more meat and particularly dairy products than before. Figure 5 shows annual per capita consumption of different categories of food in urban households between 1985 and 2003 (National Bureau of Statistics of China 1996, 1997, 2001, 2004). Over this period cereal consumption almost halved, while poultry and milk consumption increased rapidly. In 1990, annual per capita milk consumption in urban China was 4.6 kg as against 0.6 kg rurally (Fuller and Beghin 2004). By 2003 this figure had risen to 18.6 kg, although overall it was still among the lowest in the world (Fuller and Beghin 2004).

**Figure 6:**
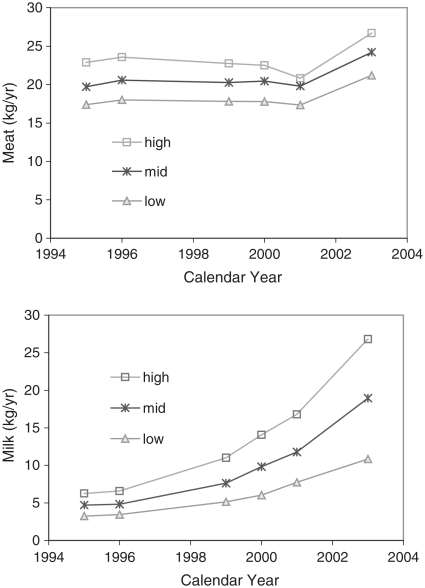
Per capita annual purchases of meat and milk of urban households in China by income level by year.

Western quick service restaurants (QSR) such as KFC and McDonalds have expanded rapidly in China since 1987, when the first KFC store opened in Beijing. By 2003 KFC had 700 QSRs in 150 cities, with over 100 in Shanghai alone (KFC Newsroom 2003; AsiaInfo Services 2003). They are popular among young people, which has disproportionately increased dietary fat intake in children (Hawkes 2008). The economic reforms in China have also dramatically increased the gap between rich and poor. In the West overweight is more common in poor families, but this appears not to be the case in China (Wang et al. 2002). High-income households spend more on food (National Bureau of Statistics of China 1996, 1997, 2001, 2004), and Figure 6 shows trends of annual per capita household consumption of milk and meat by year and income group in urban China. The ratio of consumption of meat and milk in high- to low-income households is 1.5 and 2.5, respectively, and milk intake is rising particularly fast in high-income families.

In conclusion we have documented the distribution of BMI in Chinese urban children in 2000, and have shown striking differences between the sexes, between the four Eastern cities, over time and compared to the West. Obesity is developing in China in a different way from the Western pattern, where child obesity appeared about a generation later than in adults. In China children, and particularly boys, are leading the obesity epidemic while their parents are much thinner (Wu 2006), and this has important implications both for its aetiology and for the health of Chinese children now and in the future.
